# Lectin-like oxidized low-density lipoprotein receptor-1 facilitates metastasis of gastric cancer through driving epithelial-mesenchymal transition and PI3K/Akt/GSK3β activation

**DOI:** 10.1038/srep45275

**Published:** 2017-03-27

**Authors:** Can Li, Jie Zhang, Hao Wu, Lili Li, Caiting Yang, Shushu Song, Peike Peng, Miaomiao Shao, Mingming Zhang, Junjie Zhao, Ran Zhao, Weicheng Wu, Yuanyuan Ruan, Lan Wang, Jianxin Gu

**Affiliations:** 1 Department of Biochemistry and Molecular Biology, School of Basic Medical Sciences, Fudan University, Shanghai 200032, P.R.China; 2 Key Laboratory of Glycoconjugate Research Ministry of Public Health, School of Basic Medical Sciences, Fudan University, Shanghai 200032, P.R.China; 3 Institutes of Biomedical Sciences, Fudan University, Shanghai 200032, P.R.China; 4 Department of General Surgery, Zhongshan Hospital, Fudan University, Shanghai 200032, China

## Abstract

Lectin-like oxidized low-density lipoprotein receptor-1 (LOX-1) is a pattern recognition receptor that plays a critical role in vascular diseases and host immune response. Recently, our research discovered that LOX-1 could facilitate the uptake of dying cells and cross-presentation of cellular antigen via binding with heat shock proteins, which have a close relationship with gastric neoplasia. Therefore, we speculated that LOX-1 may serve as an oncogene in gastric cancer (GC) development and progression. In this study, through immunohistochemistry staining assay and cancer-related databases, we found that LOX-1 expression was up-regulated in GC tissues and correlated with a poor prognosis in GC patients. The expression of LOX-1 was an independent prognostic factor for OS in GC patients, and the incorporation of LOX-1 with TNM stage is more accurate for predicting prognosis. Additionally, *in vitro* study by transwell assay and western blot analysis confirmed that LOX-1 could promote the migration and invasion of GC cells by driving epithelial-mesenchymal transition and PI3K/Akt/GSK3β activation. Taken together, we first explored the expression profiles, clinical significance and biological function of LOX-1 in GC, and these data suggest that LOX-1 may represent a promising prognostic biomarker for GC and offer a novel molecular target for GC therapies.

Lectin-like oxidized low-density lipoprotein receptor-1 (LOX-1) was first identified as a major receptor of ox-LDL that mainly expressed in endothelial cells^1^, macrophages^2^, smooth muscle cells and monocytes^3^. The expression of LOX-1 was up-regulated in atherosclerosis^4^, hyperlipidemia^5^ as well as diabetes^6^, and all these pathological conditions were associated with high risk of cancer development. Strikingly, the expression of LOX-1 has been demonstrated to be strongly implicated in carcinogenesis^7^,^8^. Activation of LOX-1 promotes transformation, motility and growth of several cancer cells, while down-regulation of LOX-1 can prevent such processes^9^. Moreover, some studies have shown that LOX-1 overexpression is a significant prognosis of tumor progression in advanced-stage prostate cancer^10^, colorectal cancer^11^ and squamous non-small cell lung cancer^12^.

Recently, we discovered that LOX-1 could facilitate the uptake of dying cells as well as cross-presentation of cellular antigen via binding with heat shock protein 60, 70, 90^13^. Heat shock proteins (HSPs) are originally identified as stress-responsive proteins and serve as molecular chaperones in different intracellular compartments^14^. As molecular chaperones, the main biological function of HSPs is preventing protein accumulation and facilitating refolding or elimination of misfolded proteins. It has been reported that HSPs were constitutively overexpressed in cancer cells and played vital roles in cancer cells’ survival^15^. And several lines of evidence suggest that HSPs are highly expressed in human gastric cancer (GC) and are closely associated with gastric neoplasia^16^,^17^,^18^,^19^. Therefore, we speculated that LOX-1 may be also an important molecular in GC development and progression.

GC is one of the most aggressive gastrointestinal malignancies and the third cause of global cancer deaths^20^. The only curative treatment for early-stage GC at current is adequate surgical resection, while most GC patients are diagnosed with advanced stage, which need to be combined with systemic chemotherapy^21^. However, the whole quality of life after treatment with chemotherapy is impaired and the overall survival (OS) remains low. Thus, it is necessary to search for promising prognostic markers for early-stage GC and provide novel molecular targets for GC therapeutic intervention.

Here, the expressional profiles of LOX-1 in GC tissues and the relationship between LOX-1 expression and the clinicopathologic features of GC patients were analyzed based on 100 GC cases and online databases. And we found that a high expression of LOX-1 was associated with a poor prognosis in GC patients and the prognostic accuracy of the tumor node metastasis (TNM) stage was improved as incorporation with LOX-1 expression. Besides, by using transwell assay and western blot analysis *in vitro*, we found that LOX-1 promotes migration, invasion and epithelial-mesenchymal transition (EMT) of GC cells through activating PI3K/Akt/GSK3β pathway.

## Results

### LOX-1 expression is up-regulated in gastric cancer

**Figure 1:**
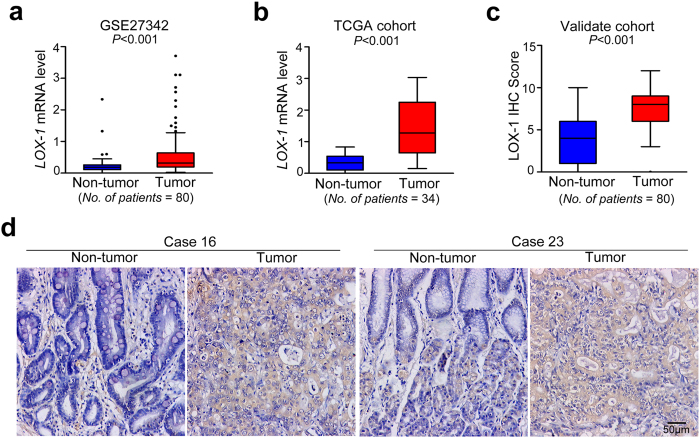
LOX-1 expression level is increased in gastric cancer. (**a**) The expression levels of LOX-1 mRNA in tumor tissues and adjacent non-tumor tissues of gastric cancer patients from GSE27342 database. (**b**) The expression levels of LOX-1 mRNA in gastric cancer tissues and paired non-tumor tissues from gastric cancer patients in TCGA cohort. (**c**) Quantitative analysis of IHC staining for LOX-1 in gastric tumor/peritumor samples from validate cohort. (**d**) Images shown are representative in 2 cases. Data are mean ± SD.

To investigate whether LOX-1 expression is correlated with GC development and progression, we first analyzed the two independent mRNA microarray analyses from GSE27342 database and TCGA cohort, and surprisingly found the mRNA expression of LOX-1 was significantly up-regulated in the GC tissues in both GSE27342 database (0.638 ± 0.789 vs 0.234 ± 0.293; *P* < 0.001; Fig. 1a) and TCGA cohort (1.411 ± 0.850 vs 0.340 ± 0.239; *P* < 0.001; Fig. 1b). And for validating the results from database, we performed immunohistochemistry (IHC) staining assay in tissue microarrays (TMAs) of 80 pairs of GC samples. As shown in Fig. 1d, the positive staining of LOX-1 was observed in the cytoplasm and the membrane of intratumor cells. Compared with peritumor normal gastric tissues, the expression level of LOX-1 protein was higher in tumor tissues (7.713 ± 2.301 vs 3.975 ± 2.728; *P* < 0.001; Fig. 1c).

### LOX-1 expression is associated with pathological characteristics and overall survival in patients with gastric cancer

**Figure 2:**
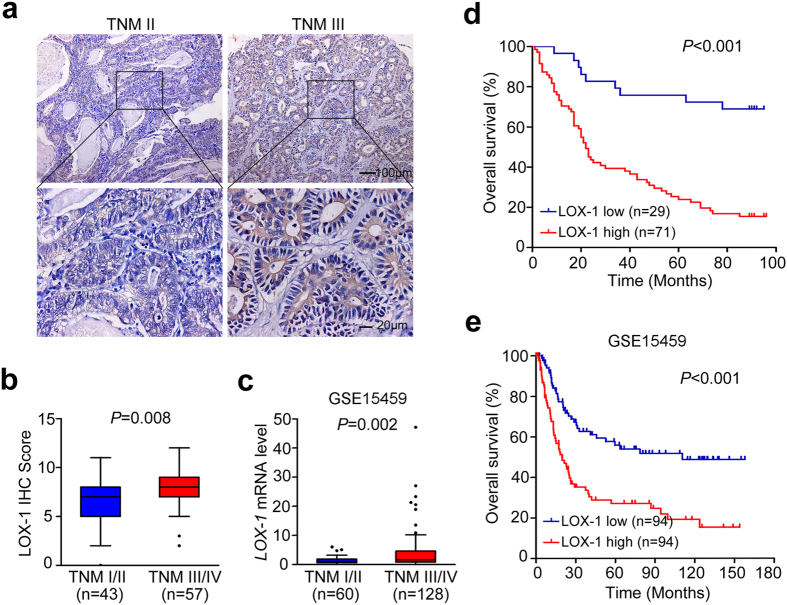
LOX-1 expression is associated with TNM stage and OS. (**a**) The representative IHC staining images of early-stage caner (TNM II) and advanced-stage cancer (TNM III) in gastric cancer patients are shown. (**b**) Quantitative analysis of LOX-1 expression in TNM I/II stage and TNM III/IV stage. Data are mean ± SD. (**c**) Quantitative analysis of LOX-1 mRNA expression in TNM I/II stage and TNM III/IV stage of gastric cancer patients from GSE15459 database. Data are mean ± SD. (**d,e**) Kaplan–Meier survival analysis of overall survival in 100 gastric cancer patients (**d**) and GSE15459 database (**e**) based on LOX-1 expression level.

In order to explore the clinical significance of LOX-1 expression in GC, we first determine the optimal cutoff value of composite expression score (CES) for IHC staining by using receiver operating characteristic (ROC) curve analysis. As shown in Supplementary Fig. S1, the optimal cutoff value of CES is 7 (CES7), and the area under the ROC curve is 0.757 (95% CI, 0.646–0.849; *P* < 0.001). CES < 7 indicated low LOX-1 expression, while CES > = 7 indicated high expression of LOX-1. Then we evaluated the correlation between LOX-1 expression and clinicopathologic features in GC patients through statistical analysis. All 100 patients were included in this study as described in Table 1, and the high expression of LOX-1 in the patients with GC was strongly associated with higher invasion depth (*P* = 0.016), lymph node metastasis (*P* = 0.021), and TNM stage (*P* = 0.006). Also, the expression level of LOX-1 was obviously increased in advanced-stage cancer (TNM III) compared with early-stage cancer (TNM II) (Fig. 2a), and the expressional difference of LOX-1 in TNM III/IV stage and TNM I/II stage was statistically significant (7.965 ± 2.138 vs 6.744 ± 2.381; *P* = 0.008; Fig. 2b), which was further confirmed by GSE15459 database (3.819 ± 6.059 vs 1.378 ± 1.179; *P* = 0.002; Fig. 2c). Additionally, Kaplan-Meier analysis of OS in 100 GC patients revealed that the patients with high expression of LOX-1 showed shorter OS than patients with low LOX-1 expression (*P* < 0.001; Fig. 2d), which was consistent with the data from database GSE15459 (*P* < 0.001; Fig. 2e). And for the 100 GC patients, the OS of patients with high LOX-1 expression was significantly shorter than those with low LOX-1 expression in both early-stage cancer (*P* = 0.046; Supplementary Fig. S2a) and advanced-stage cancer (*P* < 0.001; Supplementary Fig. S2b).

### High LOX-1 expression is an independent predictor of poor prognosis in patients with gastric cancer

**Figure 3:**
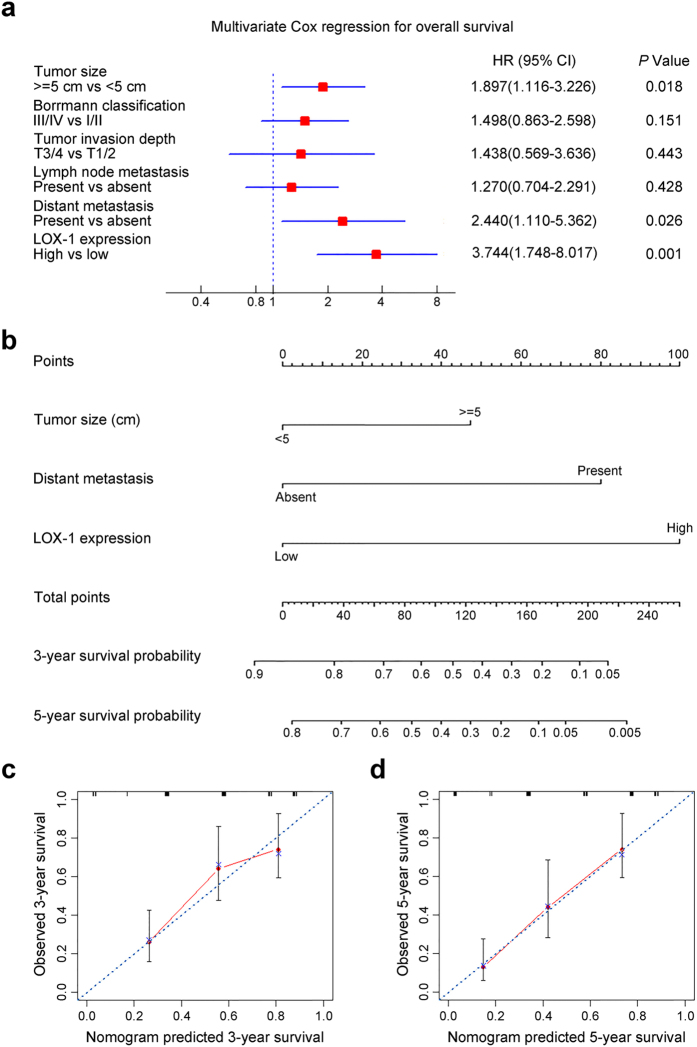
Multivariate Cox regression analysis for independent prognostic factors and nomogram for predicting 3- and 5-year overall survival in patients with gastric cancer. (**a**) Multivariate Cox regression analysis identified the independent prognostic factors for overall survival of patients with gastric cancer. (**b**) Nomogram for predicting clinical outcomes integrated LOX-1 expression (Low/High) with tumor size (<5 cm/ > = 5 cm) and distant metastasis (Absent/Present). In the nomogram, higher total point predicts worse prognosis. Addition of tumor size, distant metastasis and LOX-1 expression for each patient correspondingly gives the total point. (**c**) Calibration plot for nomogram predicted and observed 3-year survival rate. (**d**) Calibration plot for nomogram predicted and observed 5-year survival rate. Line of dashes: ideal model; vertical bars, 95% confident interval.

Univariate analysis was employed to estimate the clinical factors that influence OS of patients with GC. As described in Table 2, high LOX-1 expression was identified as a risk factor of poor prognosis (HR, 3.386; 95% CI, 2.068–5.544; *P* < 0.001). In addition, tumor size (HR, 1.774; 95% CI, 1.099–2.864; *P* = 0.019), borrmann classification (HR, 1.903; 95% CI, 1.170–3.096; *P* = 0.01), tumor invasion depth (HR, 1.988; 95% CI, 1.086–3.674; *P* = 0.026), lymph node metastasis (HR, 1.700; 95% CI, 1.035–2.793; *P* = 0.036) and distant metastasis (HR, 22.42; 95% CI, 5.881–85.43; *P* < 0.001) were risk factors for OS. To further identify the independent prognostic factors for OS in patients with GC, all risk factors for OS identified by univariate analysis were underwent multivariate Cox regression analysis. As shown in Fig. 3a, LOX-1 expression (HR, 3.744; 95% CI, 1.748–8.017; *P* = 0.001), tumor size (HR, 1.897; 95% CI, 1.116–3.226; *P* = 0.018), along with distant metastasis (HR, 2.440; 95% CI, 1.110–5.362; *P* = 0.026) were identified as independent prognostic factors for OS in patients with GC.

### Predictive nomogram for survival of patients with gastric cancer

According to the independent prognostic factors selected by multivariate Cox regression analysis, nomogram for predicting 3-year and 5-year survival in patients with GC was performed (Fig. 3b). The three points of predictors included tumor size, distant metastasis and LOX-1 expression were added to the total point, and higher total point predicted worse prognosis. For internal validation, the calibration curves for nomogram predicted 3-year and 5-year OS rates performed well with the ideal model (Fig. 3c,d).

### Combined LOX-1 expression with TNM stage improved prognosis model

**Figure 4:**
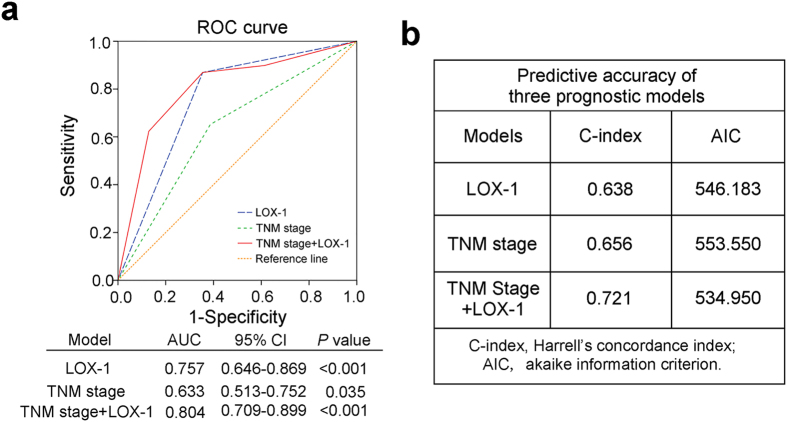
ROC analyses of prognosis for overall survival in patients with gastric cancer. (**a**) Receiver operating characteristic (ROC) curve analysis of the sensitivity and specificity for the prognosis of overall survival by LOX-1 expression model, TNM stage model and TNM stage/LOX-1 expression combination model. (**b**) The Harrell’s concordance index (C-index) and Akaike information criterion (AIC) analysis of the comparison of the predictive accuracies of LOX-1 expression, TNM stage and TNM stage/LOX-1 expression combination model.

To further improve the prognostic system for patients with GC, we constructed a prognostic model combining LOX-1 expression and TNM stage, a conventional prognostic system. ROC curve, Harrell’s concordance index (C-index) and Akaike information criterion (AIC) analysis were performed to compare the prognostic accuracy of the combination model or LOX-1 expression and TNM stage alone. As presented in Fig. 4a, the combination of LOX-1 expression and the TNM stage showed more effective prognosis (AUC, 0.804; 95% CI, 0.709–0.899; *P* < 0.001) than LOX-1 expression (AUC, 0.757; 95% CI, 0.646–0.869; *P* < 0.001) or TNM stage (AUC, 0.633; 95% CI, 0.513–0.752; *P* = 0.035) alone. These results were further confirmed by using C-index and AIC analysis. The C-index of combination model was improved to 0.721 compared with that of LOX-1 expression (0.638) and TNM stage (0.656) alone, while the AIC of combination model was decreased to 534.95 compared with that of LOX-1 expression (546.183) and TNM stage (553.55) alone (Fig. 4b).

### LOX-1 promotes the migration, invasion and epithelial-mesenchymal transition in gastric cancer cells

**Figure 5:**
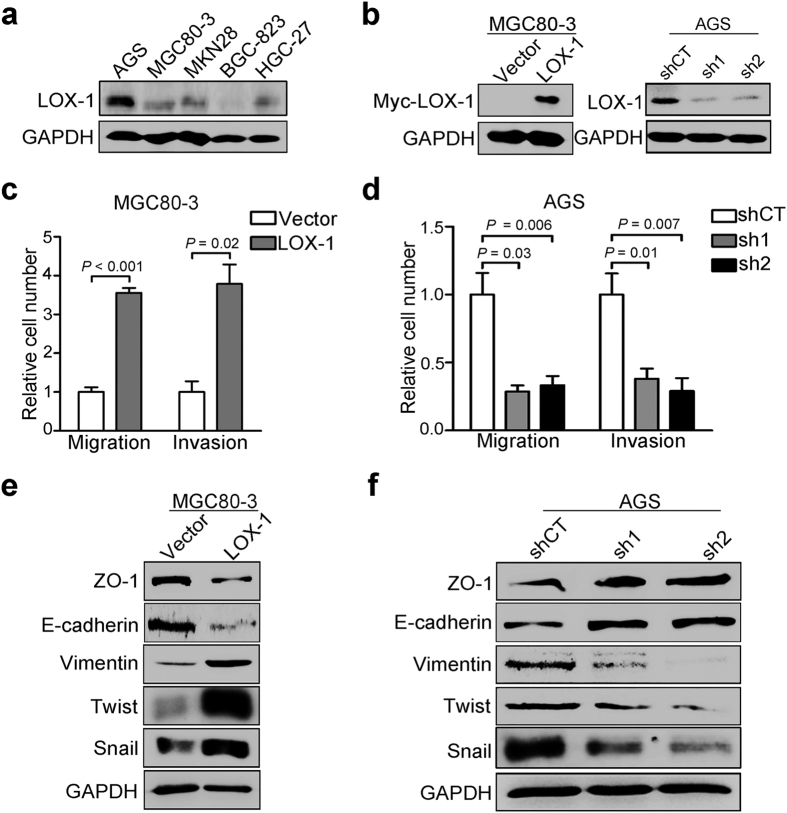
LOX-1 promotes migration, invasion and EMT in gastric cancer cells. (**a**) The expression of LOX-1 in various gastric cancer cell lines were detected by western blotting. (**b**) The expression of LOX-1 in MGC80-3 cells transfected with vector or LOX-1 overexpressing plasmid (Left panel) and AGS cells stably expressing LOX-1-specific shRNA (Right panel) were detected by western blotting. (**c**) Transwell migration and matrigel invasion assays were conducted in MGC80-3 cells transfected with vector or LOX-1. Data are mean ± SD. (**d**) Transwell migration and matrigel invasion assays were conducted in AGS cells stably expressed control shRNA (shCT) or two LOX-1-specific shRNA (sh1/sh2). Data are mean ± SD. (**e,f**) The expression of epithelial markers ZO-1 and E-cadherin, mesenchymal marker Vimentin and transcriptional repressors Snail and Twist were detected by western blotting in LOX-1-overexpressing MGC80-3 cells (**e**) and LOX-1-knock-down AGS cells (**f**).

We next evaluated the effect of LOX-1 on the migration and invasion of GC cells. As shown in Fig. 5a, LOX-1 was differentially expressed in a panel of GC cell lines. MGC80–3 cells with low basal expression of LOX-1 were transfected with LOX-1 overexpression plasmids, and AGS cells with high basal expression of LOX-1 were selected to generate stable cell lines that stably expressing LOX-1-specific small-hairpin RNAs (shRNAs) (Fig. 5b). Transwell migration and matrigel invasion assays were conducted to investigate the migration and invasion of GC cells. The results showed that the cells on the underside of the filters were increased more than three fold in LOX-1-overexpressing cells (Fig. 5c), and decreased in LOX1-knock-down cells obviously (Fig. 5d), compared with the control respectively. Given that EMT is an initial step of cancer cells migration and invasion^22^, we performed western blot analysis to examine the effect of LOX-1 on EMT-related hallmarks and transcriptional repressors. Compared with the control, epithelial markers ZO-1 and E-cadherin were down-regulated in LOX-1-overexpressing cells and up-regulated in LOX-1-knock-down cells; whereas the mesenchymal marker Vimentin and transcriptional repressors Snail and Twist were up-regulated in LOX-1-overexpressing cells and down-regulated markedly in LOX-1-knock-down cells respectively (Fig. 5e,f).

### The promoted effects of LOX-1 are associated with PI3K/Akt/GSK3β activation

**Figure 6:**
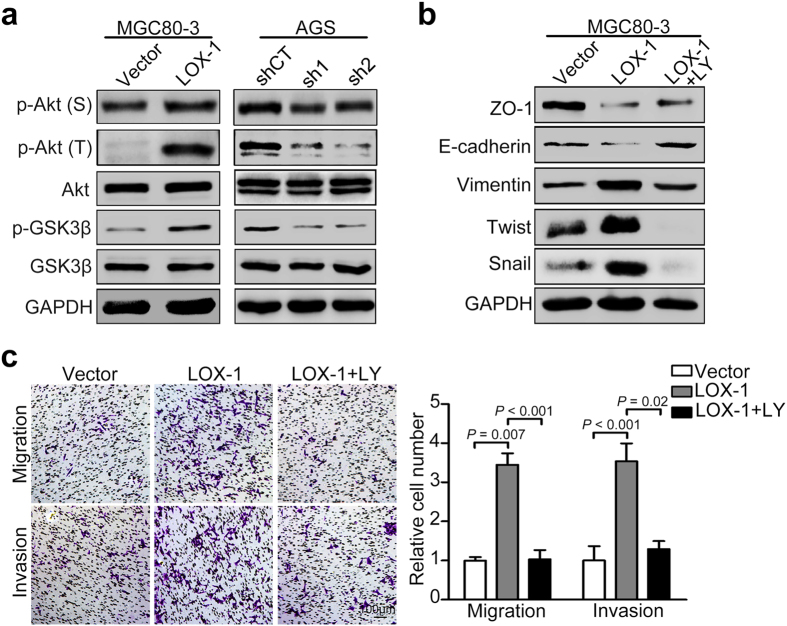
LOX-1 promotes migration, invasion and EMT of gastric cancer cells through activating PI3K/Akt/GSK3β pathway. (**a**) The western blot analysis was performed to detect the expression of Akt, GSK3β, phospho-Akt (Ser473 and Thr308) and phospho-GSK3β (Ser9) in LOX-1-overexpressing MGC80-3 cells and LOX-1-knock-down AGS cells. (**b**) MGC80-3 cells transfected with vector or LOX-1 were treated with or without LY294002, and the expression of ZO-1, E-cadherin, Vimentin, Snail and Twist were detected by western blotting. (**c**) MGC80-3 cells transfected with vector or LOX-1 were treated with or without LY294002 (LY), and applied to transwell analysis. Images shown were representative in each group. Data are mean ± SD.

To investigate the mechanism of promoted effect of LOX-1 on migratory and invasive ability of GC cells, western blot analysis was employed to examine the phosphorylation of Akt and GSK3β. We found that the phospho-Akt (Ser473 and Thr 308) and phospho-GSK3β (Ser9) levels were significantly increased in the LOX-1-overexpressing cell lines (MGC80-3). In contrast, knock-down of LOX-1 in AGS cells inhibited Akt and GSK3β activation (Fig. 6a). These results suggest that LOX-1 enhances the phosphorylation of Akt and GSK3β. To further examine whether the PI3K/Akt/GSK3β pathway was involved in the above effect of LOX-1, we treated the GC cells with an inhibitor of PI3K/Akt/GSK3β pathway, LY294002. The results showed LY294002 inhibited LOX-1-induced down-regulation of epithelial markers E-cadherin and ZO-1 and up-regulation of mesenchymal marker Vimentin and transcriptional repressors Snail and Twist (Fig. 6b). Furthermore, the transwell assay revealed that LY294002 suppressed the increased migratory and invasive ability of GC cells induced by overexpression of LOX-1 (Fig. 6c). Taken together, these results suggest that LOX-1 promotes migration and invasion as well as EMT in GC cells through activating PI3K/Akt/GSK3β pathway.

## Discussion

The present study demonstrated that LOX-1 expression was significantly increased in GC tissues and associated with invasion depth, lymph node metastasis, TNM stage and OS. Furthermore, high LOX-1 expression could be identified as an independent predictive factor for poor prognosis in patients with GC. Strikingly, combination of LOX-1 expression with TNM stage could provide much more accurate prediction for GC prognosis. Moreover, *in vitro* studies found LOX-1 promoted migration, invasion and EMT of GC cells through PI3K/Akt/GSK3β pathway. All these results indicate that the LOX-1 expression level has significant clinical relevance with GC and may represent a promising prognosis factor for GC.

Epidemiological studies have reported that patients with metabolic disorders showed increased risk of cancer incidence^23^,^24^,^25^. Under physiological conditions, LOX-1 expression is minimal but can be induced in a variety of metabolic disorders, such as atherosclerosis^4^, hyperlipidemia^5^, and diabetes^6^. Moreover, activation of LOX-1 with its ligand ox-LDL could produce additional ROS, which played vital roles in pathological processes of cancers as well as metabolic disorders^26^,^27^. In this study, we found LOX-1 was up-regulated in malignant tumors compared with adjacent normal gastric tissues, suggesting that LOX-1 may not only involve in GC tumorigenesis, but potentially served as a robust linkage among ROS, metabolic disorders and GC development. And further studies are warranted to investigate their relationship and the functional mechanism.

By IHC assay and cancer-related databases, we found that high LOX-1 expression was associated with deep invasion depth, severe lymph node metastasis, advanced TNM stage and short OS. Given the pivotal role of cancer cells migration and invasion in cancer progress, we performed transwell assay to evaluate the effect of LOX-1 on the migratory and invasive potential of GC cells *in vitro*. We found that LOX-1 promotes GC cells migration and invasion, which is consistent with previous studies conducted in other types of cancer cells^9^,^11^,^28^. It has been known that EMT plays important role in the invasive and metastatic processes of cancer cells^29^. Moreover, activation of LOX-1 by ox-LDL could promote EMT in NRK52E cells^30^. Therefore, we examined the related proteins in EMT and found LOX-1 facilitated EMT in GC cells, suggesting LOX-1 promoted GC cells migration and invasion through enhancing EMT. The PI3K/Akt/GSK3β pathways have been identified to regulate EMT^31^,^32^, metastasis^33^ and the progression of various cancers. In this study, we found LOX-1 overexpression activated PI3K/Akt/GSK3β pathways in GC cells; unexpectedly the effects of LOX-1 on cell migration and invasion activities as well as EMT were blocked by LY294002. Taken together, the results indicate that LOX-1 promotes the invasion and migration of GC cells via PI3K/Akt/GSK3β-mediated EMT. Additionally, by binding to ox-LDL, LOX-1 could increase the expression of several proangiogenic factors such as vascular endothelial growth factor and matrix metalloprotease^34^,^35^,^36^, which could contribute to tumor growth, invasion and metastasis^37^,^38^. These findings indicate another mechanism that LOX-1 may also enhance tumor angiogenesis to facilitate cancer metastasis.

Recent studies have shown that high LOX-1 expression was a significant prognostic factor in various cancers, such as advanced-stage prostate cancer^10^, colorectal cancer^11^ and squamous non-small cell lung cancer^12^. In the present study, we suggest that the LOX-1 expressed in tumor tissues was an independent prognostic factor for OS in GC patients, providing the additional evidence that LOX-1 expression can be a promising prognostic factor in different types of cancers. Moreover, although higher levels of LOX-1 was detected in advanced-stage (TNM III/IV) than in early-stage (TNM I/II) tumors, the early- or advanced-stage GC patients with low LOX-1 expression all have longer OS than those with high LOX-1 expression, indicating that controlling LOX-1 expression may be a promising target for GC treatment regardless of TNM stage. Except for its membrane receptor form expressed on cell surface, a circulating soluble form of LOX-1 (sLOX-1) has also been identified^39^. The release of sLOX-1 could be stimulated by some inflammatory factors such as interleukin 18^40^, C-reactive protein^41^, and its elevation in human serum has been considered as an important biomarker for early detection of acute coronary syndrome^42^,^43^, as well as rheumatoid arthritis^44^. Therefore, further deep study about soluble LOX-1 expression in GC is needed, as it may offer a promising non-invasive marker for GC diagnosis.

In conclusion, our research demonstrates that LOX-1 is overexpressed in GC tissues and correlates with poor clinical outcomes. By incorporation with LOX-1 expression, the prognostic accuracy of TNM staging system can be greatly improved. In addition, LOX-1 enhances the migratory and invasive ability of GC cells through activating PI3K/Akt/GSK3β pathway. These results suggest that LOX-1 may represent a promising prognosis factor for GC and serve as a novel molecular target for GC therapies.

## Materials and Methods

### Patients

For tissue microarray detection, 80 pairs of gastric tumor/peritumor samples and additional 20 tumor samples were collected from 100 GC patients who underwent surgery between July 2006 and April 2007, at Department of General Surgery, Zhongshan Hospital (Fudan University, Shanghai, P.R.China). All the selected patients were consecutively enrolled if they met the criterion of having pathologically proven GC, judged before surgery to be potentially curable, were aged less than 80 years, and were in an adequate physical condition with no serious co-morbid cardiorespiratory or renal disease. The patients were excluded if they had other malignant tumor before, or histories of adjuvant or neo-adjuvant therapies including targeted therapies. Clinicopathological data, including age, gender, tumor size (longest diameter), tumor location, borrmann classification, differentiation, and TNM stage, were collected from medical history for each patient. Tumor stage was reassessed according to the seventh edition of the American Joint Committee on Cancer TNM classification. All the patients were followed up until September 2014 with a median follow-up time of 37 months. OS was defined as the time from the date of surgery to the date of death or last visit. All methods were approved by the research medical ethics committee of Fudan University and were carried out in accordance with the approved guidelines. Informed consent on the use of clinical specimens was obtained from all patients.

### Cell lines and reagents

The human GC cell lines AGS, MGC80-3, BGC-823, HGC-27, and MKN28 were obtained from the Cell Bank of Type Culture Collection of Chinese Academy of Sciences (Shanghai, China), and cultured in RPMI-1640 or Dulbecco’s modified Eagle medium (Sigma-Aldrich, St Louis, MO, USA) supplemented with 10% fetal bovine serum (Gibco, Grand Island, NY, USA) at 37 °C in a humidified atmosphere containing 5% CO_2_. Rabbit anti-human LOX-1 polyclonal antibody and horseradish peroxidase (HRP)-conjugated anti-GAPDH antibody were purchased from Proteintech (Chicago, IN, USA). Anti-Myc antibody was obtained from Merck Millipore (Billerica, MA, USA). Anti-ZO-1, -E-cadherin, -Vimentin, -Snail, -Akt, -GSK3β, -phospho-Akt (Ser473/Thr308) and -phospho-GSK3β (Ser9) antibodies were obtained from Cell Signaling Technology (Beverly, MA, USA). Anti-Twist antibody was from Santa Cruz Biotechnology (Santa Cruz, Dallas, TX, USA). The PI3K inhibitor, LY294002 was purchased from Selleck Chemicals (Houston, TX, USA).

### TMAs, IHC staining and evaluation

The TMAs were constructed from formalin-fixed, paraffin embedded surgical specimens, and LOX-1 IHC staining was performed using UltraVision Quanto Detection System (Thermo Scientific, Fremont, CA, USA). In brief, the slices were baked at 65 °C for 6–8 h and deparaffinized using xylene and rehydrated using alcohol. Then, antigen retrieval was implemented by heating the tissue slices in a microwave oven for 20 min in boiling citrate buffer solution. Subsequently, the slices were immersed in UltraVision Hydrogen Peroxide Block (Thermo Scientific) for 10 min to eliminate endogenous peroxidase, and then blocked unspecific protein using UltraVision Protein Block (Thermo Scientific). The tissue sections were then incubated with anti-LOX-1 antibody (Proteintech Group, Inc., dilution 1:200) at 4 °C overnight. The next day, the slices were incubated with Primary Antibody Amplifier Quanto (Thermo Scientific) and HRP Polymer Quanto (Thermo Scientific) for 10 min, respectively, and then immunoreactive signals were developed using DAB Quanto (Thermo Scientific). Negative control received an identical treatment except the primary antibody and showed no LOX-1 positive signal.

The photographs were obtained by Nikon eclipse Ti-s microscope (Tokyo, Japan) and assessed by two investigators who had no knowledge of the patients’ clinical data to exclude subjectivity. For IHC results assessment, a previous scoring method was used^45^. CES with full range from 0 to 12 was performed to show the staining intensity and frequency of positive cells.

### TCGA and GEO databases

The databases used in this study are publically available from the Cancer Genome Atlas TCGA and the GEO database (accession number: GSE27342^46^, GSE15459^47^). For the TCGA database, gene expression dataset was downloaded by using the UCSC Cancer Browser (https://genome-cancer.ucsc.edu/proj/site/hgHeatmap/), and 34 pairs of gastric tumor/peritumor samples were analyzed. For the GEO databases, the relative mRNA expression was achieved through the Oncomine database (https://www.oncomine.org/resource/login.html).

### Plasmid construction, transfections, and stable cell lines

The cDNA encoding LOX-1 was obtained by PCR and cloned into the pcDNA 3.1/myc-His(-)A vector (Invitrogen, Carlsbad, CA, USA) to generate pcDNA3.1-LOX-1-Myc/His construct. The shRNA plasmid targeting LOX-1 and scramble control shRNA were purchased from Shanghai Genechem Co., LTD (Shanghai, China). Transient transfections were carried out using Lipofectamine 3000 (Invitrogen) following the manufacturer’s protocol. For stably knocking down LOX-1, cells were infected with LOX-1 shRNA lentiviral particles, and stable cell lines were selected with puromycin (0.5 μg/mL). In some experiments, cells were treated with LY294002 (50 nM) for 12 h before harvest.

### Western blotting

Protein from cell lysates were separated by SDS–polyacrylamide gel electrophoresis, transferred onto polyvinylidene difluoride membranes (Millipore), and incubated with primary antibodies, followed by incubation with horseradish peroxidase (HRP)-conjugated secondary antibody (Santa Cruz Biotechnology). The immunoreactive proteins were visualized by using enhanced chemiluminescence detection kit (Tiangen Biotech, Beijing, China) and the image analyzer ImageQuant LAS 4000 (GE Healthcare, Abingdon, UK).

### Transwell assay

The effects of LOX-1 on the migratory and invasive abilities of GC cells were assayed in BD Falcon 12-well plates with Transwell inserts containing 8-μm pore filters (Millipore). Approximately 1 × 10^5^ cells were seeded for migration assay and invasion assay. For invasion assays only, the bottom of transwell chamber was coated with BD Matrigel Basement Membrane Matrix (BD Biosciences, San Diego, CA, USA). Briefly, transfected cells or stable cell lines were resuspended in 500 μL serum-free medium and seeded into the upper chamber of the assay system. The bottom wells of the system were filled with 1500 μL complete growth medium. Migration or invasion of cells were determined 24 h or 48 h later, respectively. Migrated or invaded cells were washed twice with ice-cold phosphatebuffered saline (PBS) and then fixed with 4% paraformaldehyde for 15 minutes and stained with 0.1% crystal violet (Beyotime Institute of Biotechnology, Jiangsu, China) for 15 minutes. Cells on the upper side of the filters were removed with cotton-tipped swabs, and the filters were washed in PBS. Cells on the underside of the filters were viewed and counted under a microscope. Each experiment was repeated 3 times.

### Statistical analysis

Statistical analyses were performed with SPSS 22.0 (SPSS Inc., Chicago, IL), Prism software (GraphPad, La Jolla, CA), and R software version 3.2.5 with the “rms” package (R Foundation for Statistical Computing, Vienna, Austria). ROC curve analysis was used to determine the optimal cutoff value for CES and compare the prognostic accuracy for combination model. Pearson’s chi-squared test and Fisher’s exact test were applied for categorical variables; continuous variables were analyzed by the Student’s t test. Survival and univariate analysis were determined by Kaplan-Meier analysis, and the significance of the difference between curves was calculated with the log-rank test. The Cox proportional hazards regression model was applied to perform multivariate analysis. Nomogram was generated by R software with “rms” package. The prognostic accuracy was also measured by calculating the C-index and AIC. All statistical analyses were two-sided, and *P* value < 0.05 was considered statistically significant.

## Additional Information

**How to cite this article**: Li, C. *et al*. Lectin-like oxidized low-density lipoprotein receptor-1 facilitates metastasis of gastric cancer through driving epithelial-mesenchymal transition and PI3K/Akt/GSK3β activation. *Sci. Rep.* **7**, 45275; doi: 10.1038/srep45275 (2017).

**Publisher's note:** Springer Nature remains neutral with regard to jurisdictional claims in published maps and institutional affiliations.

## Supplementary Material

Supplementary Information

## Figures and Tables

**Table 1:** Association between LOX-1 expression and patient characteristics in 100 gastric cancer patients.

Characteristics	All patients (N = 100)	LOX-1 expression		
		Low (N = 29)	High (N = 71)	*P* value*
Age (y)				
Mean ± SD	64.45 ± 10.21	64.52 ± 9.36	64.42 ± 10.69	0.966
Gender				
Male	64	20	44	0.509
Female	36	9	27	
Tumor size (cm)				
Mean ± SD	6.10 ± 3.61	5.31 ± 2.30	6.40 ± 4.00	0.185
Tumor location				
Proximal	13	2	11	0.558
Middle	13	3	10	
Distal	57	19	38	
Unspecific	17	5	12	
Borrmann classification				
I	7	4	3	0.374
II	39	11	28	
III	40	10	30	
IV	10	2	8	
Differentiation				
Moderately	30	10	20	0.532
Poorly	70	19	51	
Depth of invasion				
T1	8	4	4	0.016
T2	7	3	4	
T3	61	22	43	
T4	20	0	20	
Lymph node metastasis				
N0	29	12	17	0.021
N1	15	5	10	
N2	26	9	17	
N3	30	3	27	
Distant metastasis				
Absent (M0)	91	29	62	0.056
Present (M1)	9	0	9	
TNM stage				
I	10	5	5	0.006
II	33	14	19	
III	48	10	38	
IV	9	0	9	

*χ^2^ test, Fisher exact test or Student’s t test was performed.

**Table 2:** Univariate analyses for overall survival in 100 patients with gastric cancer.

Factors	Overall survival	
	HR (95% CI)	*P* value
Age (y)		
<65	Reference	0.053
> = 65	1.601 (0.993–2.580)	
Gender		
Female	Reference	0.484
Male	0.838 (0.508–1.379)	
Tumor size (cm)		
<5	Reference	0.019
> = 5	1.774 (1.099–2.864)	
Tumor location		
Proximal + middle	Reference	0.070
Distal + unspecific	0.624 (0.348–1.119)	
Borrmann classification		
I + II	Reference	0.010
III + IV	1.903 (1.170–3.096)	
Differentiation		
Moderately	Reference	0.079
Poorly	1.577 (0.949–2.621)	
Tumor invasion depth		
T1 + T2	Reference	0.026
T3 + T4	1.988 (1.086–3.674)	
Lymph node metastasis		
Absent (N0)	Reference	0.036
Present (N1 + N2 + N3)	1.700 (1.035–2.793)	
Distant metastasis		
Absent (M0)	Reference	<0.001
Present (M1)	22.42 (5.881–85.43)	
LOX-1 expression		
Low	Reference	<0.001
High	3.386 (2.068–5.544)	

HR, Hazard ratio; CI, Confidence interval; *P* < 0.05 was considered to be statistically significant.
