# Supplementary material for: Lectin-like oxidized low-density lipoprotein receptor-1 facilitates metastasis of gastric cancer through driving epithelial-mesenchymal transition and PI3K/Akt/GSK3β activation
Source: Sci Rep. 2017 Mar 27;7:45275. doi: 10.1038/srep45275 (PMC5366889; doi:10.1038/srep45275)
Supplement: Supplementary Information [file srep45275-s1.pdf]

Lectin-like oxidized low-density lipoprotein receptor-1 facilitates metastasis of gastric cancer through driving epithelial-mesenchymal transition and PI3K/Akt/GSK3β activation

Can Li[1, 2], Jie Zhang[1, 3], Hao Wu[1, 2], Lili Li[1, 2], Caiting Yang[1, 2], Shushu Song[1, 2], Peike Peng[1, 2], Miaomiao Shao[1, 2], Mingming Zhang[1, 2], Junjie Zhao[4], Ran Zhao[3], Weicheng Wu[1, 2], Yuanyuan Ruan[1, 2], Lan Wang[1, 2*], Jianxin Gu[1, 2, 3]

Supplementary Information

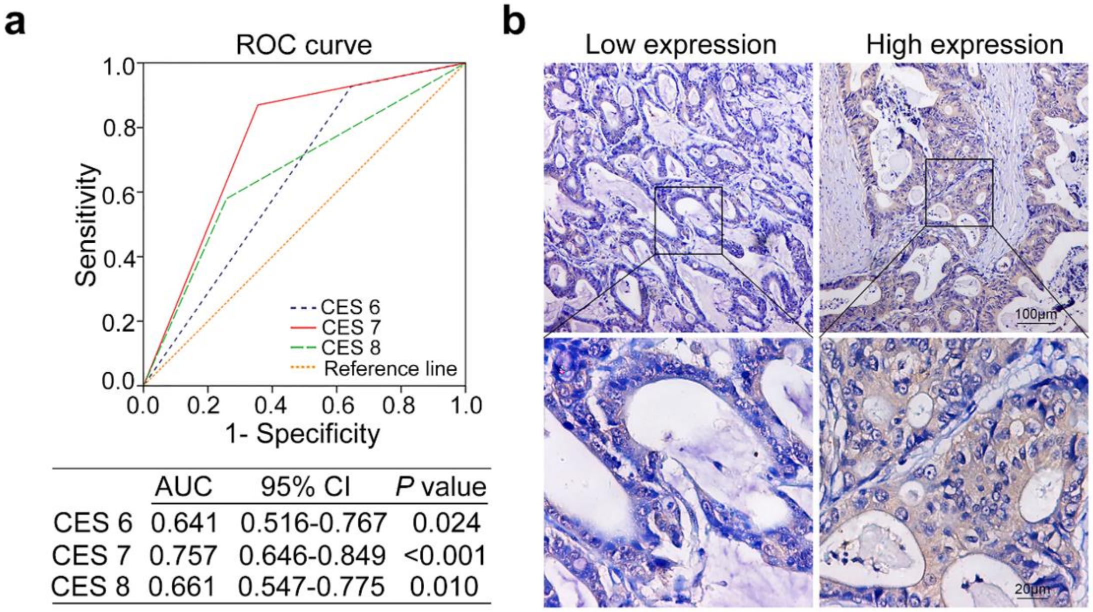

Supplementary Figure S1. The ROC curve analyses of LOX-1 immunohistochemistry staining. (**a**) Receiver operating characteristic (ROC) curve analyses of different cutoff values of composite expression score (CES), and the area under the ROC curve (AUC), 95% confident interval (95% CI) and P value are shown. (**b**) Representative images show low LOX-1 expression (Left panel) and high LOX-1 expression (Right panel) in gastric cancer tissues.

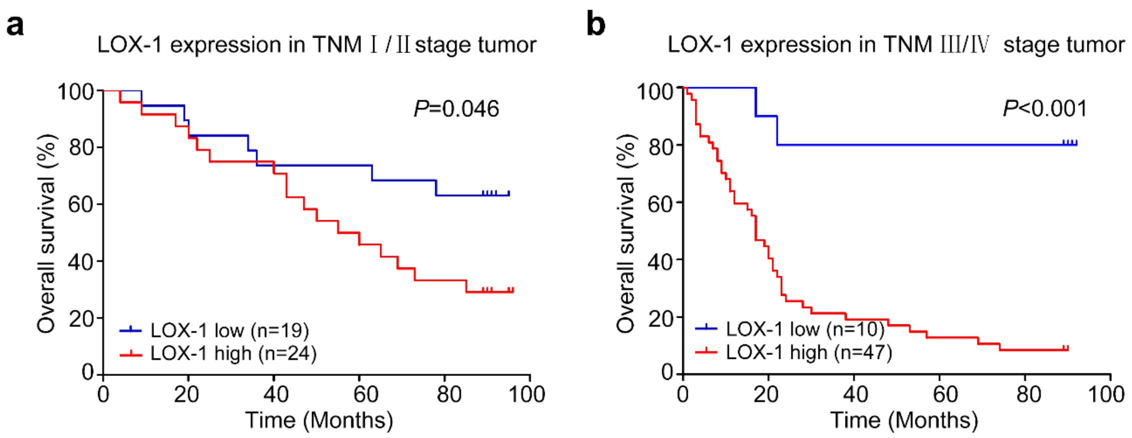

Supplementary Figure S2. Subgroup analyses of overall survival for patients with gastric cancer according to LOX-1 expression. Kaplan–Meier analyses of overall survival were performed in gastric cancer patients with early-stage cancer (TNM I/II) (**a**) and advanced-stage cancer (TNM III/IV) (**b**).
